# Supplementary material for: Decoding the causal drivers of spatial cellular topology
Source: iScience. 2026 May 24;29(6):115794. doi: 10.1016/j.isci.2026.115794 (PMC13224383; doi:10.1016/j.isci.2026.115794)
Supplement: Document S1. Figure S1 and Table S1 [file mmc1.pdf]

iScience, Volume *29*

Supplemental information

Decoding the causal drivers of spatial cellular topology

Prannav Shankar, Huan Liang, Uthsav Chitra, and Rohit Singh

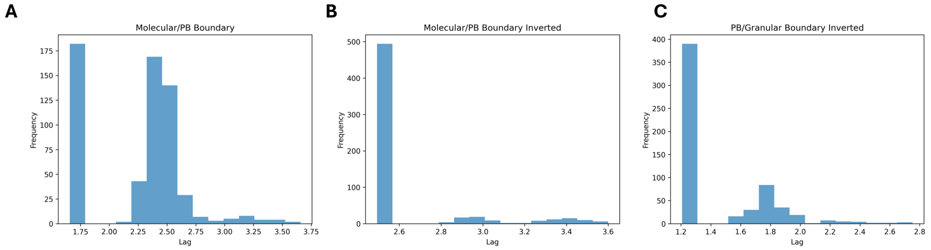

Figure S1: **Distribution of lags learned by GLACIER.** (Related to STAR Methods) Distribution of lags identified by GLACIER across all candidate interactions in **(A)** the boundary between the molecular and Purkinje-Bergmann (PB) layers using the isodepth d, **(B)** the boundary between the molecular and PB layers using the inverted isodepth d', and **(C)** the boundary between the PB and granular layers using the inverted isodepth d'.

Table S1: Key Resource Table

REAGENT or RESOURCE	SOURCE	IDENTIFIER
Deposited data		
Slide-SeqV2 of mouse cerebellum available from Cable et al, Nature Methods (2022)		`https://singlecell.broadinstitute.org/single_cell/study/SCP1663`
Software and algorithms		
Our code is available at `https://github.com/rohitsinghlab/glacier`		DOI: 10.5281/zenodo.19500378
